# Habitat Selection of a Large Carnivore along Human-Wildlife Boundaries in a Highly Modified Landscape

**DOI:** 10.1371/journal.pone.0086181

**Published:** 2014-01-23

**Authors:** Chihiro Takahata, Scott Eric Nielsen, Akiko Takii, Shigeyuki Izumiyama

**Affiliations:** 1 Interdisciplinary Graduate School of Science and Technology, Shinshu University, Nagano, Japan; 2 Department of Renewable Resources, University of Alberta, Edmonton, Alberta, Canada; 3 Education and Research Center of Alpine Field Science, Faculty of Agriculture, Shinshu University, Nagano, Japan; Università degli Studi di Napoli Federico II, Italy

## Abstract

When large carnivores occupy peripheral human lands conflict with humans becomes inevitable, and the reduction of human-carnivore interactions must be the first consideration for those concerned with conflict mitigation. Studies designed to identify areas of high human-bear interaction are crucial for prioritizing management actions. Due to a surge in conflicts, against a background of social intolerance to wildlife and the prevalent use of lethal control throughout Japan, Asiatic black bears (*Ursus thibetanus*) are now threatened by high rates of mortality. There is an urgent need to reduce the frequency of human-bear encounters if bear populations are to be conserved. To this end, we estimated the habitats that relate to human-bear interactions by sex and season using resource selection functions (RSF). Significant seasonal differences in selection for and avoidance of areas by bears were estimated by distance-effect models with interaction terms of land cover and sex. Human-bear boundaries were delineated on the basis of defined bear-habitat edges in order to identify areas that are in most need of proactive management strategies. Asiatic black bears selected habitats in close proximity to forest edges, forest roads, rivers, and red pine and riparian forests during the peak conflict season and this was correctly predicted in our human-bear boundary maps. Our findings demonstrated that bears selected abandoned forests and agricultural lands, indicating that it should be possible to reduce animal use near human lands by restoring season-specific habitat in relatively remote areas. Habitat-based conflict mitigation may therefore provide a practical means of creating adequate separation between humans and these large carnivores.

## Introduction

As human land domination has expanded, many species have disappeared from their primary range. Nevertheless, some carnivores still survive within or near anthropogenic landscapes [1]. There are several reports of large carnivores periodically frequenting peri-farmlands: for instance, grey wolves (*Canis lupus*) [2]; bobcats (*Lynx rufus*) [3]; and American black bears (*Ursus americanus*) [4]. The inevitable consequence of the proximity of wildlife habitats to human-dominated lands is an increase in undesirable human-wildlife interactions, which in many cases involving a large carnivore can be fatal for both. The conservation of large carnivores newly adapted to human landscapes is one of the greatest challenges facing local wildlife managers because of the difficulty of reconciling the ecological requirements of animals with the need to preserve human life and property. The reduction of human-carnivore interactions is critical to the sharing of finite land, and this only seems possible through a better understanding of the processes and patterns involved in the use of human landscapes by wildlife.

Attention must first be directed to areas which animals find particularly attractive. Because only limited areas remain undisturbed in anthropogenic landscapes, animals need to find ways to derive some benefit from their habitat while simultaneously keeping their distance from the risks posed by humans. For instance, American black bears avoided frequent contact with people by shifting their core active time from day to night [4]. An intermediate level of housing density a short distance from a large forest edge was the main factor in human-bear interaction as it offered a combination of foraging opportunity and defensive refuge [5]. The effects which distance from human disturbance has on wildlife have been assessed worldwide [6]–[8]. Most previous research has been conducted on a broad scale, and there have been few studies done at a fine scale and in highly populated areas in which there is a substantial overlap with wildlife habitats.

Areas of overlap in human-wildlife habitat result in linear-shaped boundaries where both habitats are separated by distinct dichotomic geographical and ecological features (e.g. forest cover and open, flat and rugged terrain, etc.). The extent and structure of human-wildlife boundaries are an important factor in human-wildlife interaction. For example, sufficient space and environmental gradients within a boundary may give both people and animals a chance to avoid sudden and frequent contacts. In contrast, a boundary without enough space or tonal structure becomes a potential source of human-wildlife conflict. In some developing regions, such sharpening of human-wildlife boundaries has occurred due to massive land use expansion. It also happens in other regions where, conversely, land use changes by de-populated and aged rural societies cause wildlife habitat use to shift back toward the fringes of agriculture or urban areas. Increased tension is the inevitable consequence of sharpened human-wildlife boundaries. Large carnivores in particular suffer from a high risk of mortality due to the fears of local communities who have experienced fatal encounters, even if only on rare occasions. Although conflict mitigation is critical to the conservation of large carnivores in peripheral human lands, investigations of the habitats potentially associated with human-carnivore encounters have seldom been conducted [5]. Delineating naturally occurring human-wildlife boundaries also has the potential to help land use managers prioritize areas for management.

In Japan, current changes in human land use may have effects on the distribution and structure of human-wildlife boundaries. One of the more distinct land use changes has been a drastic reduction in the traditional use of coppice forests on the fringes of agricultural fields or settlements (see detail in [9]). Such secondary forests have become densely covered after the cessation of logging, and the area of unmanaged privately owned forests has reached about 30% of total forested lands [10]. Constant human presence and open patches in the secondary forests formally played an important role in preventing direct and frequent contact with wildlife. Moreover, about 11% of farmland has been abandoned since the 1960s due to a nationwide decline and the aging of the rural population [11]. For the last decade, Asiatic black bears (*Ursus thibetanus*) have experienced high mortality due to a surge in conflicts and contacts with people. In 2006, unusually large numbers of bears were sighted within and around rural and suburban lands, and as a result about 40% of the estimated total Japanese black bear population was killed [12]. In Nagano prefecture, the number of destroyed bears was 558, well in excess of the 150 estimated to be necessary to maintain viable populations [13]. This was partly due to intolerance of wildlife on the part of local communities [14], but also to an unreliable population estimate. Even though Huygens [15] reported no association between damage costs and prior-year bear kills, lethal control is still the major management method in Japan. A precautionary principle should be applied to Asiatic black bears in Japan because carnivore populations are seriously impacted by social intolerance and the prevalence of lethal control [16], [2]. Under such circumstances, a reduction in the frequency of bear use near human-dominated lands is an urgent need if the number of bears killed is to be reduced.

We focused on habitat selection by Asiatic black bears near human landscapes to identify the key factors potentially associated with frequent contact and conflicts with humans. First, using GPS bear location data by sex-season groups, we examined how bears responded differently depending on the season to distance from roads, forest edges and rivers on the assumption that such linear landscape features influenced the shape of human-bear boundaries. We hypothesized that strongly selected variables during summer compared with autumn would be the key factors relevant to conflicts because summer is the peak season of human-bear conflict. Second, we estimated resource selection functions (RSF) to predict the distribution of relative probability of bear selection for each sex-season group. Finally, we delineated the human-bear boundaries by defining the edges of both bear habitat and human-dominated land. It was expected that this study would provide local wildlife managers with spatial models that help prioritize the location of management needs including the re-establishment of buffer functions and the development of adequate pre-avoidance schemes. This research aimed to explore a habitat-based approach to conflict mitigation to achieve co-existence with these elusive large carnivores occupying the fringes of human-dominated land.

## Materials and Methods

### Ethics Statement

Asiatic black bears were captured and fitted with a collar equipped with a global positioning system (GPS) (Televilt, Lindesberg, Sweden). All animals were handled in accordance with the “The Mammal Society of Japan Guidelines for the Treatment of Animal Samples (2009)” drafted with reference to the guidelines issued by the Institutional Animal Care and Use Committee (IACUC). For all locations used in the capture, release and tracking of bears, permission was granted by the Nagano Prefectural Office, the Ministry of the Environment and the District Forest Office.

### Study Site and Landscape Covariates

**Figure 1:**
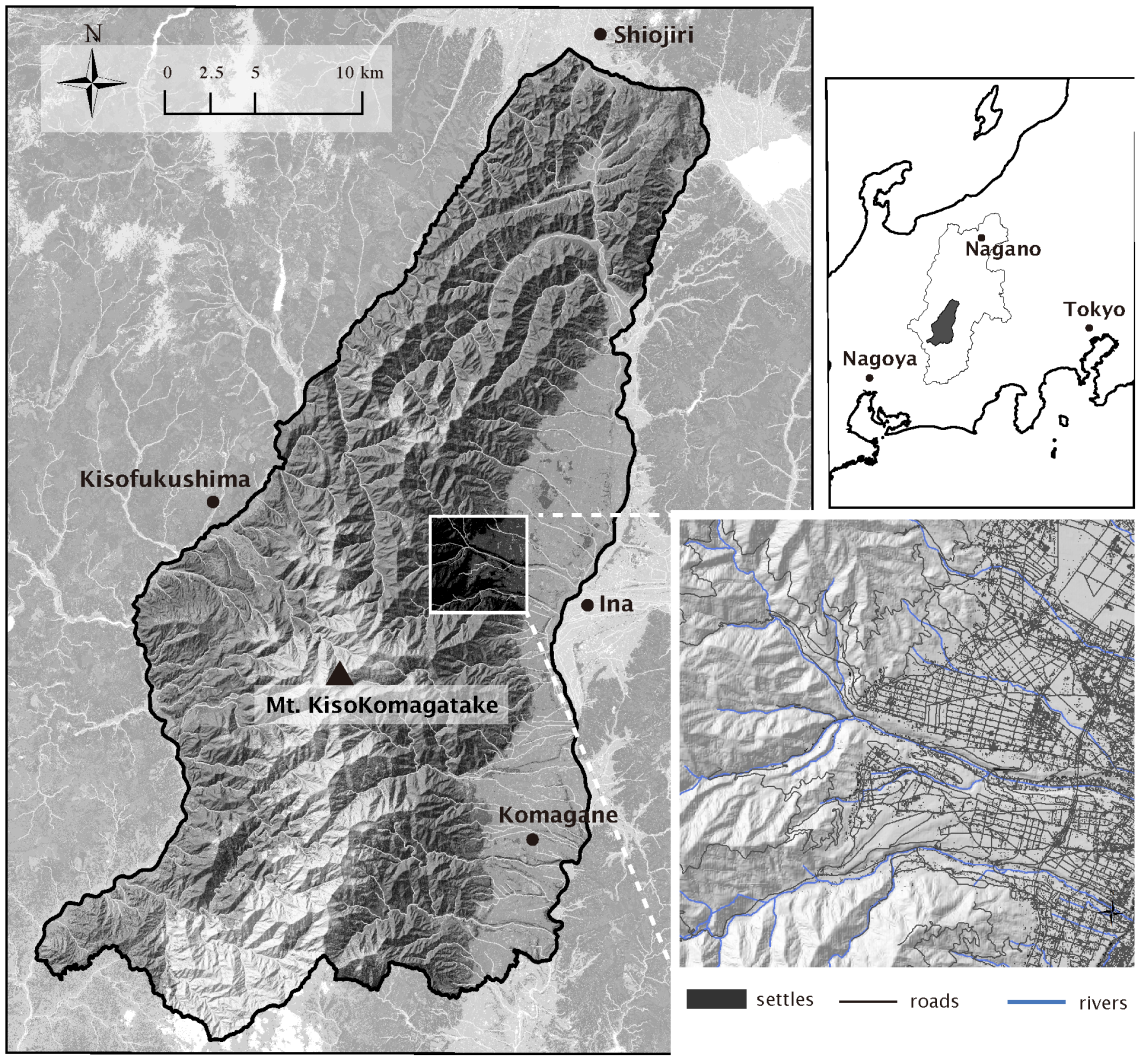
The study area of 1,023^2^ (35°48′27N, 137°49′47E) including the central Japan Alps located between Ina and Kiso valley, the southern part of Nagano prefecture in the Honshu island of Japan. The example section (bottom right) has a typical distribution of the landscape features that proved most important in the production of human-bear boundary maps.

Our research area (Figure 1) consists of two major landscapes with distinct configurations. One is a range of rugged mountain covered by various forest types including native subalpine coniferous forest, temperate broad-leaved deciduous forest and monocultural plantations converted from native deciduous forests. The other is valley basin dominated by farmlands and urban infrastructure with a highly dense road network (7.74 km/km^2^, elevation<900 m). In the foothills, there extend unmanaged secondary-growth forests that were once coppice forests. Additionally, several riparian forests running across the human matrix connect the foothills and urban areas.

Landscape features relevant to Asiatic black bear habitat and anthropogenic lands were reclassified into 9 land cover types from a satellite image taken on 22^nd^ June, 2007 (ALOS-AVNIR2) by a supervised mapping technique using Multispec v 3.2.1© [17]. The land cover categories consisted of 5 types of forest cover, 1 open-forest, 2 human-dominated landscapes and others (Table 1). Generally, a forest has diverse structures depending on its successional stage, and can provide different foraging opportunities for bears. For this reason, we generated a forest age map from the polygon layers on the Basic Planning maps produced by the National and Regional Forestry departments, and we reclassified 10 indices according to the age of the forests by referring to the historical records of forest management in the Basic Planning maps.

**Table 1:** Landscape covariates considered to influence habitat selection by Asiatic black bears in the central Japan Alps. 9 land cover types were reclassified (6 types of vegetation, 2 human-landscape classes and others) from a satellite image with 10 m resolution.

Landscape Covariate	Description (Original data source, data type (unit) and range; % = a proportion of total study area)	Variable code
Land cover	Categorical	land cover
Larch plantation	Plantation of Larches *Larix leptolepis* (0 or 1), 26.2%	larch_pt
Coniferous plantation	Plantation of Japanese Cedars *Cryptomeria japonica* or Cypress *Chamaecyparis obtusa* (0 or 1), 7.3%	conifer_pt
Broadleaved forest	Forest of Deciduous broadleaved trees *Fagaceae*, *Rosaceae*, *Cornaceae*, *Juglans*, *Castanea* etc. (0 or 1), 13.7%	deciduous_f
Red pine forest	Forest of Japanese red pine *Pinus densiflora* (0 or 1), 7.4%	redpine_f
Open regenerating	Canopy openings with various regeneration stages consist of shrub and herbaceous plants (0 or 1), 5.3%	open_g
Subalpine forest	Forest mainly consists of evergreen conifers in subalpine zone (0 or 1), 6.2%	alpine_f
Farmlands	Multiple crop lands and livestock sheds (0 or 1), 14.6%	farmland
Near town	Paddy fields, villages and towns (0 or 1), 6.7%	neartown
Others	Alpine meadow, permanent snow, rocks, water surface and clouds (0 or 1), 10.6%	others
Forest Age	10 indices (1–10) from 0 to over 110 years old reclassified from the Basic planning maps of National and Regional Forestry	frstAge
Topographic features	Linear	
Elevation	Measured from 10 m DEM (m), mean 1268, range 456–2956	elev
CTI	Compound Topographic Index, range 0.4–21.9	cti
Roughness	Terrain Ruggedness Index, range 0–9.0	roughness
Solar Radiation		
Summer	Represented at 1st August, (Wh/m2), range 1087.8–7298.8	solar
Autumn	Represented at 1st October, (Wh/m2), range 115.6–5022.4	
Distance variables	Linear	
Forest edges	Distances from the nearest edge of forest cover (m), range 0–1000	frstEdg
Rivers	Distances from the nearest river (m), range 0–2686.8	rivers
Roads in open lands	Distances from the nearest road in open lands (m), range 0–6967.1	road_o
Roads in forests	Distances from the nearest road in forests (m), range 0–3284.9	road_f

To explore how Asiatic black bears are affected by abiotic conditions, we created terrain raster predictors using various GIS algorithms applied to a 10 m Digital Cartographic Data Standards digital elevation model (DEM). Given the high ratio of mountains in our study area, terrain roughness was generated by calculating a square root of standard deviation of the DEM using the roughness tool in the Geomorphometric and Gradient Metrics Tool [18]. According to the general principles of ecology, conditions of sunlight and water are the major factors determining a plant community's potential to affect local wildlife food distribution. To include a variable of potential water content of soils, a compound topographic index (CTI) was developed by combining slope and flow accumulations calculated from the DEM. The degree of incoming solar energy for each pixel was computed by employing the solar radiation analysis tool in a spatial analyst extension (ArcInfo9.3©) capable of taking into account the effects of latitudinal and local terrain uniqueness on insolation. We set two days as representative for each season, 1st August for summer and 1st October for autumn. Four distance variables were estimated: roads in open land (open roads), roads in forest cover (forest roads), forest edges and rivers. We then measured the nearest distances to each bear location. To create the forest-edge map, we reclassified the 9 land cover types into 2 major land cover types, open and forested lands. Using both the edge-enhancing and smoothing filters in the Neighborhood tool in the spatial analyst extension, we delineated the border lines between open and forest lands as forest edges. All maps were prepared as rasters at a 10 m resolution to facilitate the raster calculations necessary to develop and apply an RSF value to each pixel (Table 1).

### Animal location data and sampling design

We used hourly GPS locations collected from 24 bears (14 females and 10 males) during both summer and autumn in 2008–2011. We defined summer as the period from 1st July to 10th September, and autumn as being from 11th September to denning according to dietary pattern correlated with seasonal shift. Resource selection functions (RSF) were estimated following a designIII(1) as suggested by Manley et al [19] where individual animals are identified with used resource units defined by telemetry data and available resource units randomly chosen from within the home ranges of individual animals. We delineated MCP (the minimum convex polygon) as individual home ranges derived from the GPS relocation data for each bear, and sampled 1000 points randomly from each MCP to yield the available resource units on the assumption that all resources within the MCP would be available to the bear during a season. The landscape attributes of the used units were contrasted with those of the available resource units using logistic regression to model habitat selection by Asiatic black bears.

### Investigation of spatial distance effects

On the assumption that summer-specific habitat selection would be relevant to the frequency of human-bear interactions, we focused on seasonal differences in probability of bear selection in terms of distance from roads, rivers and forest edges using logistic regression based on used-available locations as the binary response variable. Starting with a model for the single explanatory variable of continuous distance (D) as the base line, we manually structured a distance-effect model by stepwise addition of quadratic terms of distance (D^2^), season (SS), sex (SX), land cover (LC) and three interactions with season (D:SS, SS:SX, SS:LC). Log-likelihood, AIC (Akaike's information criteria) and Δ*_i_* values were used as measures for the selection of the final distance-effect model for each linear landscape. The final model was used to investigate which of the covariates had substantial effects on the differences between summer and autumn by predicting the odds ratio of selection by Asiatic black bears (Table 2).

**Table 2:** Comparison between distance-effect models using logistic regression with distance variables for each linear landscape features partly including interaction terms for seasons according to log-likelihood (LL), AIC, and AIC score as changes in AIC from the lowest model (Δ*i*).

Model	Road_open_dist			Road_forest_dist			Forest_edge_dist			River_dist		
	LL	AIC	Δ*_i_*	LL	AIC	Δ*_i_*	LL	AIC	Δ*_i_*	LL	AIC	Δ*_i_*
D+D^2^+D:SS+SS:LC+SS:SX	−58865	117777	0	−58943	117931	0	−59296	118638	0	−59780	119607	0
D+D^2^+D:SS+SS:LC	−58878	117799	22	−58967	117976	45	−59316	118675	37	−59794	119631	24
D+D^2^+D:SS+LC	−60092	120193	2416	−59889	119791	1859	−60415	120842	2204	−60929	121871	2264
D+D^2^+D:SS+SS:SX	−60150	120315	2538	−59925	119863	1932	−60452	120919	2281	−60977	121968	2361
D+D^2^+D:SS+SX	−60152	120197	2420	−59928	119869	1938	−60457	120925	2287	−60982	121977	2370
D+D^2^+D:SS	−60155	120320	2544	−59939	119888	1957	−60462	120934	2296	−60985	121980	2373
D+D^2^+SS	−60465	120938	3161	−60054	120117	2186	−61198	122405	3767	−61168	122344	2737
D+D^2^	−60709	121423	3647	−60216	120439	2508	−61340	122687	4049	−61306	122617	3010
D	−60721	121447	3670	−60627	121259	3327	−61344	122692	4054	−61322	122648	3041

The variable names: continuous distance (D), quadratic terms of distance (D^2^), season (SS), sex (SX), land cover (LC) and three interactions with season (D:SS, SS:SX, SS:LC).

The logit-odds of the final distance-effect model was calculated by using the following equation:
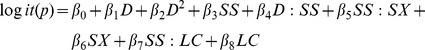
(1)where *p* was probability of bear selection, and *ß* was a coefficient. And the logit-odds for bear selection during summer (season = 1) relative to autumn (season = 0) were given by the equation,
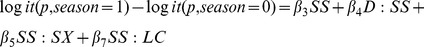
(2)The aim of the distance-effect analysis was to determine which land cover types were significantly responsible for the large differences between the two seasons with respect to bear selection. In order to yield the odds-ratio between seasons, we ran models for each sex-land cover combination by controlling the interaction terms of the categorical variables SS:SX and SS:LC in equation (2) applying the coefficients ß_5_ and ß_7_. A total of 64 models (8 land cover: 2 sex for each linear landscape) were developed to estimate the odds ratio by adjusting the mean value of the continuous distance variable. We selected models containing land cover types that influenced the first and second largest differences between summer and autumn according to the odds ratio, their absolute differences being calculated by subtraction from 1 ( = no selection) with elimination of the sign, and the significant level measured by the Wald test (*p*<0.001). Finally, we set 6 specific distances from 0 to 2000 m and predicted a mean probability of bear use at each distance to assess how much variation in seasonal response by bears would be explained by land cover variables as distances changed.

### Habitat model structure and validation

Since effects of distance decline rapidly as bear locations occur beyond the linear landscape feature, we transformed distance variables into exponential decay functions, e^−α*d*^ where –α was the decay constant and *d* was distance (m) from the linear landscape. [20]. Quadratic terms in continuous predictors, except the non-linear exponential decay variables, were included if necessary, and interaction terms potentially relevant to foraging habitat and anthropogenic factors were added. Prior to variable selection, co-linearity among linear predictors was checked, and variables correlated with more than three other variables were removed. For model selection, we employed a mixed effect logistic regression with random intercept to balance the disproportional number of observations among individual animals [21]. To develop a plausible model in terms of bear biology, we conducted univariate logistic regression and a manual step-forward procedure [22]. After determining the full variable sets suitable to each sex-season group model, we constructed a global model incorporating all the variables in order to make comparisons among sex-season groups to determine how the predictive human-bear (HB) boundaries differed.

We built an individual RSF model separately for each bear, and averaged the coefficients within the sex-season group. We expected that disproportional error rates would be caused by the variety of sample sizes in some variables among individual animals, and that this would have substantial effects on the averaged coefficients. For example, the reliability of coefficients for individual bears were not equal when sample sizes in a land cover type differed significantly among the bears. To deal with these imbalances, we used an inverse variance weighted method to obtain appropriate averages that incorporated the differences in standard error for each parameter estimate. [20]. Next, we applied the mean coefficients to each predictor of GIS layers to develop RSF habitat maps. A reclassifying tool with quantile breakpoint was used to rank the RSF values into 10 classes to represent the spatial distribution of relative probability of habitat selection by Asiatic black bears across the target research area. To assess the credibility of the predictive performance of our RSF models, we prepared 2490 GPS observations for testing as samples independent of the training data used for model building. We first calculated the utilization function (*Ux_i_*) for each predicted RSF class. The mid-points of the RSF values were multiplied by the area of each RSF class, and divided by the total value to obtain the *Ux_i_* for each class [23]. The total number of the test data was multiplied by the *Ux_i_* to determine the expected frequencies fell within each RSF class. And then, using a linear regression, we contrasted the expected frequency and observed frequency to assess the significant level of the slope by R^2^ and χ^2^ goodness of fit test [23].

### Human-Bear (HB) boundary delineation

We identified HB boundaries by employing an edge-detection technique based on a focal statistical tool involving a moving window GIS operation. First, we combined farmlands, paddy fields, human-settled areas and roads into one landscape layer that represented “human lands”. Second, we created bear-habitat edges by transforming the RSF maps with a circle-shaped moving window that determined the value of each pixel from the sum of its surrounding pixels. The generated bear-habitat maps were classified into three types (0 = others, 1 = sharp edge, 2 = moderate edge) with sharp edge defined by RSF classes 9 and 10, and moderate edge by RSF classes 7 and 8, respectively. In the same manner, the edges of human lands were detected and classified into two types (0 = others, 1 = edge). The edges of both bear habitat and human lands were determined by using a moving window circle with a 6-pixel radius (60 m) on the basis of animal movement. Here, we used the median step length (sequential distance between each location), which was about 70–100 m for the 4 groups of Asiatic black bears in our study. Finally, we multiplied the edges of bear habitat and human lands to generate 2 ranked boundary areas. The boundary was classified into two types (1 = sharp HB boundary, 2 = moderate HB boundary) by performing a raster calculation with the two edge layers as follows: (habitat edge; 0, 1, 2)×(human land edge; 0, 1). The non-overlapping edges were then zeroed out (0×0, 0×1 and 0×2) to leave only pixels of human-edge equal to 1 (1×1 and 1×2). All our GIS work was performed on ArcGIS (ESRI v9.3©) and statistical work was done in Stata (SE v12.0, College Station, Texas).

## Results

We collected a total of 44,652 bear locations across sex-season groups (F-summer, N = 10297, mean 792±278 SD; F-autumn, 14357, 1044±411; M-summer, 9788, 979±430; M-autumn, 10210, 1021±456). On average, female MCP home ranges (summer, mean 21.96±40.83 km^2^ SD; autumn, 24.45±25.72 km^2^) were smaller than those of males (summer, 38.52±44.44 km^2^; autumn, 99.55±107.64 km^2^; t-test on the paired two samples of females and males, *p* = 0.008, *df* = 19), and the MCP ranges of most bears included human-dominated lands (F-summer, 9/13 = 9 of 13 bears; F-autumn, 10/13; M-summer, 10/10; M-autumn 9/10).

### Distance effects of linear landscapes on bear responses

Asiatic black bears showed significant seasonal differences in their responses to distances from open roads, forest roads, rivers and forest edges (e.g. χ^2^ = 1107, *df* = 2, *p*<0.001 in a comparison of Road_open_dist models between D+D^2^ and D+D^2^+D:SS). The manually constructed model (stepwise variable entry) revealed that the land cover covariate had a substantial influence on model fit for all distance variables, and the inclusion of season and land cover interaction (SS:LC) dramatically improved models indicated by Δ*_i_* which is the difference in AIC between models (e.g. χ^2^ = 2197, *df* = 15, *p*<0.001 in comparison of Forest_edge_dist models between D+D^2^+D:SS+LC and D+D^2^+D:SS+SS:LC) (Table 2). As a result, for all linear landscapes we selected the final distance-effect model with the lowest AIC that contained quadratic terms of distance and season inclusive of all interactions. We then used the models to investigate the seasonally varied response of Asiatic black bears to distance from the linear landscapes.

Table 3 illustrates the odds ratio yield by equation (2) using coefficients of the selected distance-effect models that resulted in an effect size for each of the land cover covariates on a seasonal difference in the probabilities of selection by Asiatic black bears. As shown in the distance-effect models for open roads, the absolute difference in the odds ratio between the two seasons was the greatest in the model with alpine forest as a land cover covariate, followed by the model with open regenerating lands and deciduous forest (*p*<0.001). The other three distance-effect models revealed significant seasonal changes associated with deciduous forest and red pine forest (Table 3). In addition, we observed a significantly positive red pine forest selection in summer relative to autumn and, conversely, a significantly positive selection of deciduous forests in autumn relative to summer regardless of sex difference.

**Table 3:** Differences between summer and autumn depending on changes of land cover types and sex in response to distances from open roads, forest roads, forest edges and rivers by Asiatic black bears in the central Japan Alps.

		females			males		
model	land cover	Odds Ratio	P>|z|^a^	Diff.Season^b^	Odds Ratio	P>|z|^a^	Diff. Season^b^
Road_open_dist adjusted distance at mean = 1395.42 m LRT = 5022.88, *df* = 22, *p*<0.001	larch_pt	0.750	0.000	0.250	1.126	0.000	0.126
	alpine_f	1.867	**0.000**	**0.867**	1.920	**0.000**	**0.920**
	farmland	0.838	0.003	0.162	0.861	0.012	0.139
	conifer_pt	0.961	0.454	0.039	0.988	0.826	0.012
	deciduous_f	0.511	**0.000**	**0.489**	0.526	**0.000**	**0.474**
	open_g	1.607	**0.000**	**0.607**	1.653	**0.000**	**0.653**
	neartown	0.749	0.038	0.251	0.770	0.060	0.230
	others	1.140	0.596	0.140	1.172	0.520	0.172
	redpine_f	1.238	0.000	0.238	1.273	0.000	0.273
Road_forest_dist adjusted distance at mean = 304.78 m LRT = 4868.30, *df* = 22, *p*<0.001	larch_pt	0.842	0.000	0.158	0.880	0.000	0.120
	alpine_f	1.075	0.545	0.075	1.124	0.328	0.124
	farmland	1.065	0.279	0.065	1.113	0.062	0.113
	conifer_pt	0.874	0.011	0.126	0.913	0.089	0.087
	deciduous_f	0.536	**0.000**	**0.464**	0.560	**0.000**	**0.440**
	open_g	1.190	0.017	0.190	1.244	0.003	0.244
	neartown	0.984	0.906	0.016	1.028	0.840	0.028
	others	1.587	0.064	0.587	1.658	0.042	0.658
	redpine_f	1.443	**0.000**	**0.443**	1.508	**0.000**	**0.508**
Forest_edge_dist adjusted distance at mean = 193.52 m. LRT = 4161.50, *df* = 22, *p*<0.001	larch_pt	0.867	0.000	0.133	0.933	0.010	0.067
	alpine_f	0.774	0.027	0.226	0.834	0.113	0.166
	farmland	0.763	0.000	0.237	0.822	0.001	0.178
	conifer_pt	0.885	0.019	0.115	0.952	0.355	0.048
	deciduous_f	0.583	**0.000**	**0.417**	0.628	**0.000**	**0.372**
	open_g	0.824	0.008	0.176	0.887	0.101	0.113
	neartown	0.734	0.025	0.266	0.790	0.087	0.210
	others	0.905	0.682	0.095	0.974	0.915	0.026
	redpine_f	1.262	**0.000**	**0.262**	1.358	**0.000**	**0.358**
River_dist adjusted distance at mean = 367.10 m LRT = 3192.60, *df* = 22, *p*<0.001	larch_pt	0.890	0.000	0.110	0.950	0.057	0.050
	alpine_f	1.084	0.484	0.084	1.158	0.202	0.158
	farmland	1.007	0.910	0.007	1.075	0.209	0.075
	conifer_pt	0.893	0.029	0.107	0.953	0.362	0.047
	**deciduous_f**	0.533	**0.000**	**0.467**	0.570	**0.000**	**0.430**
	open_g	1.287	0.000	0.287	1.374	0.000	0.374
	neartown	0.889	0.395	0.111	0.949	0.706	0.051
	others	1.201	0.451	0.201	1.283	0.306	0.283
	**redpine_f**	1.518	**0.000**	**0.518**	1.621	**0.000**	**0.621**

Odds ratios for bear selection were calculated on the basis of logistic regression by controlling season inclusive interaction terms with sex and land cover. LRT denotes Log-likelihood Ratio Test in comparison with the constant only model.

a denotes the level of significance by Wald statistics at the point estimate and

b was the absolute difference from no selection (odds ratio = 1).

**Figure 2:**
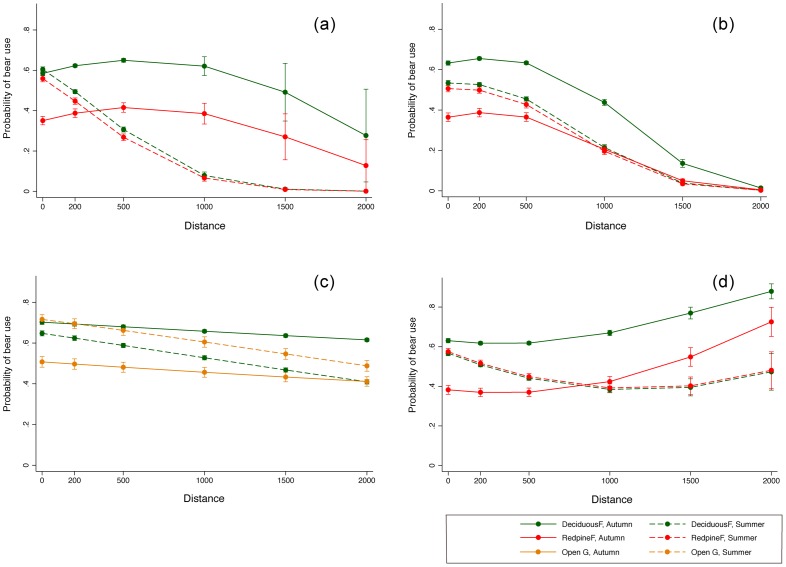
Changes in probability of use by Asiatic black bears along with increasing distances from linear landscape features; forest edges (a), forest roads (b), open roads (c) and rivers (d) in distance effect models. Dots represent the mean of probability at a distance, and error bars represent confidence intervals predicted in logistic regression as a function of the distance variables with interactions of land cover and season; summer (dashed line), autumn (continuous line), deciduous forest (green), red pine forest (red), and open regenerating lands (khaki).

Note that the odds ratio for summer versus autumn in the distance-effect models referred to above was estimated only by holding the continuous distance variables at their mean. Therefore, we predicted the changes of probability of bear use as changes in distance from the linear landscapes by estimation of the mean probability at the specific distance (Figure 2 a–d). To develop graphs of each distance-effect prediction, we used models incorporating the top two most influential land cover covariates for seasonal difference without discriminating between sexes because sex differences had a less marked effect on seasonal changes than land cover covariates (Table 2). It was assumed that predictions produced using the open-road model with alpine forest were less relevant to bear selection because of the very long distances between geographical locations, so alpine forest was excluded as a predictor.

According to the predicted forest-edge distance-effect model (Figure 2a), the probability of use by Asiatic black bears increased dramatically as distances from forest edges shortened during summer. Selection within both deciduous and red pine forests exhibited similar trends in summer, and the difference between the mean probabilities for the two forests was much smaller than the difference during autumn (1/7 difference: mean of three distances; 200 m, 500 m, 1000 m). Probability of bear use increased with increasing proximity to forest road in both seasons (Figure 2b), but the pattern varied slightly. For example, there was a dramatic decline in probability of use at a distance of 200 m to 500 m in red pine forest during summer whereas bear use within a 500 m distance was relatively stable during autumn in both deciduous and red pine forests. In the open-road model, the probability of bear use in open regenerating lands near open roads was higher during summer than in autumn, while the probability in deciduous forests near roads was lower in summer than in autumn (Figure 2c). A high probability of bear use close to rivers occurred only in summer. The difference between the two forest types was very small in summer compared with the difference in autumn, and this was similar to patterns observed in forest-edge and forest-road models (Figure 2a,b). Overall, the changes in probabilities as distance increased were more gradual in the open-road and river models (Figure 2c,d). Therefore, it seems that open roads and rivers had more extensive distance effects on selection by Asiatic black bears than forest edges or forest roads.

### Habitat Selection by Asiatic black bears

Elevation was removed from the final variable because of a close correlation with open roads (R^2^ = 0.75), forest roads (R^2^ = 0.59) and roughness of terrain (R^2^ = 0.53). CTI was excluded due to its lower contribution. According to the results from the distance-effect models, the effects of open roads and rivers seemed more extensive than those of forest roads and edges (Figure 2c,d). Therefore, we determined the decay constant α for the exponential form of distance variables to be −0.005 for open roads and rivers, and −0.01 for forest roads and edges since a smaller value for the constant yields a more gradual decay. Those values, however, were roughly set as we had observed that the range of distance effects varied depending on the land cover types (Figure 2 a–d). In the final variables selected by univariate logistic regression (Table 4), there was a significant seasonal difference among predictors. In summer, both female and male bears strongly selected areas in close proximity to forest-edge. Although forest roads, rivers and solar radiation had a relatively weak influence as single predictors, once forest edges were associated with these covariates, it provided a positive or negative leverage to remain ranked in the top 5.

**Table 4:** The final set of variables selected through univariate analysis in mixed effect logistic regression for the global RSF model across season-sex groups of Asiatic black bears; numbers indicate the rank of 15 variables ordered in accordance with Wald statistics.

	Summer				Autumn			
Variables	females		males		females		males	
frstEdg×solar	**1**	*	**2**	*	6		8	
deciduousF×frstEdg	**2**		**3**		**4**		**3**	*
frstEdg×rivers	**3**		**1**	*	**5**		7	
road_f×frstEdg	**4**	*	**4**		8		**5**	
frstEdg	**5**	*	**5**	*	12		9	
road_o	6	*	6	*	15		10	
rivers×solar	7		7		10	*	12	
land cover	8		12		**1**		**1**	
deciduousF×rivers	9	*	8		**3**		**2**	*
rivers	10	*	9	*	13		14	
frstAge+frstAge^2^	11		14		7		11	*
roughness	12	*	10	*	11	*	15	
deciduousF×frstAge	13		13		**2**	*	**4**	
road_f	14	*	11	*	14		6	
solar+solar^2^	15		15	*	9		13	

Significant mark * = p<0.05, all parameters were included the constant.

Bold letters = the top 5 ranks.

Our habitat selection models indicated that bears constantly selected deciduous forest year round (Table S1). In particular, the selection during autumn was highly significant (*p*<0.001). We found a remarkable selection of areas near forest edges during summer for both sexes. For instance, the probability of bear selection at a 20 m distance from forest edges was about two times higher than at a 100 m distance for female bears (odds 3.74/1.81), and about three times higher for male bears (odds 7.00/2.40). Distance to rivers was a strong predictor in the multivariate summer-habitat model, especially associated with forest edges (Table S1, S2). The difference between the odds ratio at 20 m and 100 m distances from rivers (Female: 3.03, Male: 7.36) increased where forest edges (20 m distance) were associated with rivers (Female: 21.45, Male: 18.01). Overall, forest-edge effects on bear selection nearly disappeared during autumn. Female bears generally avoided areas near forest roads in summer (odds 0.67 at 20 m), but selected in autumn (odds 2.26 at 20 m). In contrast, male bears selected areas near forest roads during summer (odds 1.40 at 20 m), but avoided such areas during autumn (odds 0.44 at 20 m).

### Habitat predictions and Human-Bear (HB) boundary maps

**Figure 3:**
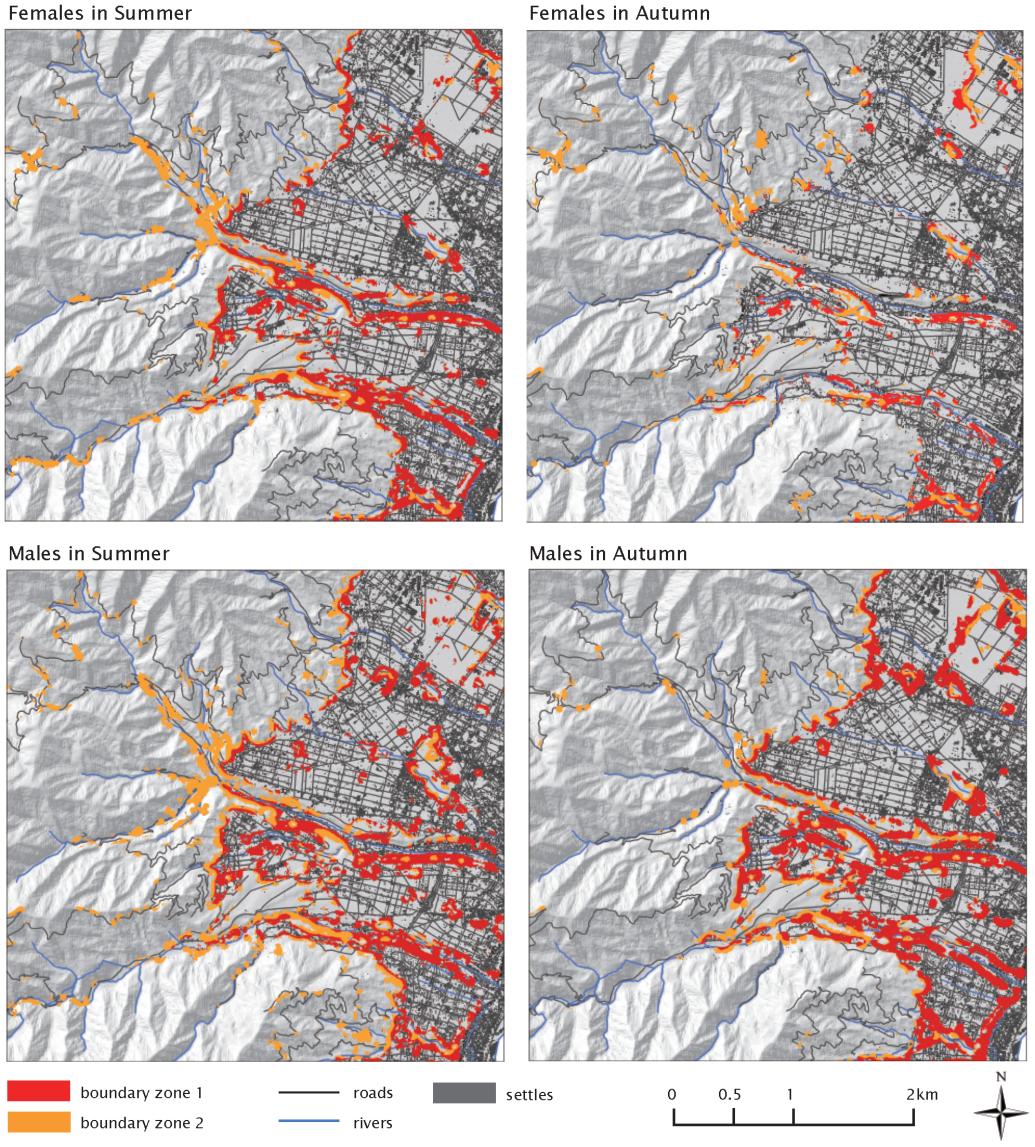
Distribution of the boundaries between humans and Asiatic black bears in the central Japan Alps (10×10 m resolution). The colors indicate sharp boundary: the overlapped edges of RSF class 9–10 and human lands (red) and moderate boundary: the overlapped edges of RSF class 7–8 and human lands (orange). The different panels show HB boundaries for females in summer (top left), females in autumn (top right), males in summer (bottom left) and males in autumn (bottom right).

Overall, the averaged coefficients from individual models were consistent with population models (Table S2). There were large error rates in the estimates of coefficients for some bears because their home ranges were exceedingly isolated from open roads. However, we confirmed that the inverse variance weights worked reasonably well to offset these unbalancing effects on the averaged coefficients for the population estimates. Our final RSF maps had acceptable predictive performances for the independent test data sets (F-summer, R^2^ = 0.84, *df* = 9, *p*<0.01; F-autumn, R^2^ = 0.93, *df* = 9, *p*<0.01; M-summer, R^2^ = 0.80, *df* = 9, *p*<0.01; M-autumn, R^2^ = 0.93, *df* = 9, *p*<0.01). The HB boundary zones were estimated for each sex-season group (Figure 3). Boundaries stretching along rivers and the fringes of foothills were a common characteristic among the 4 group models, and the extent was greater in male than female HB boundaries. The range of female-summer HB boundaries was about 28.6% of the area of high RSF classes, and it decreased to 4.3% in autumn as male HB boundaries decreased from 49.9% in summer to 10.5% in autumn. Notably, male bears exhibited a high probability of use in the HB boundary zones during summer, as was evidenced by the fact that 9.4% of test GPS locations fell within this boundary zone (Table 5).

**Table 5:** Summary of human-bear (HB) boundaries for each sex-season group of Asiatic black bears in the central Japan Alps.

		Boundary zone				bear locations
		Area of sharp boundary zone 1 (km^2^)	Area of moderate boundary zone 2 (km^2^)	Total area of boundary zone 1 and 2 (km^2^)	Boundary zone overlapped with 9–10 RSF class (%)	Test bear locations within boundary zone (%)
females	summer	30.2	32.8	63.1	28.6	8.4
	autumn	22.6	12.7	35.3	4.3	1.2
males	summer	65.3	36.8	102.1	49.9	9.4
	autumn	22.4	44.2	66.6	10.5	5.8

## Discussion

### Key habitat components relevant to human-bear interactions

Despite the common perception of bears as mature forest-interior dwellers, the frequency with which Asiatic black bears selected sites near forest edges was greater than random. Forest-edge selection has been reported in several studies on Grizzly bears [24]–[25], Scandinavian brown bears [26] and American black bears [27]. To our knowledge, this is the first report of forest-edge selection by Asiatic black bears. The importance of phenology in bear habitat selection is well attested [28]–[30]. Therefore, the great seasonal change in red pine forest and deciduous forest indicates that the availability of food may be the main reason for the selection of forest edges, which provide herbaceous and fruiting food during the most food-scarce season [31]. On the other hand, bears may use edge space as a refuge for hiding. Grizzly bears selected forest-edge areas in open regenerating lands [24], and brown bears used habitat edges for a day bed to avoid diurnal human activities [32]. In our case, Asiatic black bears preferring to stay on the edge of a red pine forest during summer run the risk of encountering people because forests are commonly located adjacent to agricultural and residential areas in this landscape. Bears are known to have complex responses to roads, and our segregation of road types successfully revealed different responses to open roads and forest roads. There was no clear avoidance of open roads, which accords with previous findings of a neutral response to high-use roads by bears [33]–[34]. However, our distance-effect model clearly demonstrated that bear response to roads differs according to the land cover near the road. Despite the differences, Asiatic black bears selected red pine forests and deciduous forests near forest roads during both summer and autumn. This might be explained by the fact that roads in forests provide surrogates for the kind of natural openings which have recently decreased in our region. Our habitat selection models support the findings of sex-season specific responses to the forest roads in earlier studies [35]–[36]. For example, subordinate individual bears such as females with cubs or young males made use of human-influenced areas [37], and adult male bears displayed a greater tolerance of using roads as a movement corridor [34]. In the case of Asiatic black bears, the greater seasonal difference found for males can be explained by the fact that their larger home range contains a relatively large portion of deciduous forest, rich in autumn food resources, at a moderate distance from forest roads.

Riverside environment strongly affected the summer habitat selection of Asiatic black bears, functioning as an ecotonal belt providing various edible plants with a combination of light and moisture (e.g. *Cardiocrinum* sp, *Petasites* sp) [31]. The selection of red pine forest can be explained by the abundant understory of fruiting shrub species, attractive to bears during the season of food shortages. The significant reduction in the selection of red pine forest during autumn may reflect the fact that there is an abundance of food such as insects, particularly ants [38], in the summer only. The forests extend continuously from the foothills to the riparian forests in the lower plains that draw bears to areas encompassed by agriculture and urban landscapes. Remnant riparian forests offer bears a linear habitat with sufficient cover for foraging and bedding [27] and a dispersal corridor for a large range of movement [39]–[40]. Despite this potential, the green corridor can undermine populations as it leads to frequent contacts with people [41]. In our study landscape, the riparian forest that ended up facing the urban fringe does not function as a corridor. Young adult males are both the largest dispersers and the group of bears with the highest mortality [10]. Therefore, the riparian forests in our region have an adverse effect on the survival of these bears by playing the role of “false dispersal corridors”.

In autumn, the land-cover variable was the best predictor in our univariate logistic regression (Table 2), and a preference for deciduous forest was prominent during the hyperphagia season due to a high correlation with the availability of fruited oak trees [38]–[39]. The substantial difference between deciduous and red pine forest was revealed by our distance-effect models in autumn. In contrast, the smaller difference between the two forests in summer implied the importance of bear food occurring in edges or understory regardless of forest type. Over all, the great seasonal differences in our habitat model may reflect the amplified temperature gradient caused by the combination of temperate climate and montane slope exerting a significant influence on phenology. On the one hand, it should be noted that our habitat models did not include variables of time of day. The importance of temporal aspects in habitat selection has been stressed by several recent studies [42], and adding temporal variables may bring an important new dimension to our understanding of how bears use peripheral human lands.

### Predicted human-bear boundaries

Our human-bear boundary maps gave a reasonably accurate picture of remarkable seasonal differences, and the patterns of bear response to linear landscape features were successfully shown. Consequently, we are confident that the largest extent of human-bear boundaries during summer corresponds to the peak season of human-bear conflicts and incidents [43]. The boundaries of male bears were similarly distributed in both summer and autumn against significant seasonal differences in the females' map. We observed that some male bears were attracted by specific croplands and stayed for longer periods after the date chosen to partition the seasons. This is why the pattern of autumn boundaries remained similar to that in the summer. During the inter-crop season, croplands producing food attractive to bears (e.g. corn, apples and other fruits) have potentially negative impacts on the seasonal migration of bears by detaining bears longer, changing their intrinsic behavior and so causing chronic conflicts [44].

### Influence of human land use changes on human-wildlife interactions

Although the negative impacts of large-scale deforestation on primary bear habitat is obvious [45]–[46], it has also been recognized that open regenerating lands after timber harvests are beneficial to American black bears [47]–[48] and Grizzly bears [24]. Brodeur [49] reported that open regenerating shrubs offer a high density of fruit plants. Likewise, patchily distributed early serial fruits were found to be an important resource for Asiatic black bears during the summer food shortage [50]–[51]. The increasing number of abandoned villages and farmlands might offer Asiatic black bears an attractive alternative to open shrub land. We were not able to distinguish between actually used and abandoned farmlands from the satellite image, and we recognize this may be the reason for the positive association of farmlands and bear habitat in our models. Additionally, red pine forest is a typical semi-natural growth after cessation of logging. Given that they are commonly located near or within human landscape, abandoned farmlands and red pine forests are key areas with respect to the need to strike a balance between the conservation of summer bear habitat and the reduction of human-bear contacts.

Bears occurring in forest edges adjacent to human settlements risk being sighted by people, and in many cases this results in bear mortality. Mountain roads in this region are used for both forestry and recreation, such as hiking, fishing and picking wild edible plants. The unexpected human presence resulting from these irregular activities can threaten bears in nearby forest edges or on forest roads and lead to tragic encounters. It is equally important to exercise caution in riparian forest areas. And there is also a high risk that croplands located near the linear green belt will change the natural behavior of bears. Given the inevitable bear use of areas near human-dominated lands during summer, pre-avoidance schemes to regulate access to the HB boundary zones should be established to minimize encounters with bears.

### Management implications and future directions

Creating spatial separation between humans and large carnivores is a challenging task for wildlife managers worldwide. One simple and efficient method is to construct physical barriers, and electric fencing around cropland has become widely used to prevent crop damage by wildlife. Another option is to create buffer zones. For example, cover can be removed by clear-cutting forest-edge shrubs to increase permeability and so deter wildlife from lingering near human lands. Such indirect means of preventing encounters by changing wildlife behavior would be effective only if implemented in the key areas predicted on the basis of reliable habitat estimates. Given the complexity of the problem, it is necessary to endeavour to change not only wildlife behavior, but also human behavior. Human security is a genuine concern for people living close to occupied carnivore habitats. There are specific areas and periods in which human-wildlife interaction becomes more likely. For example, our RSF model indicated that there is an about 80% probability that farmlands producing bear attractants within 100 m of a forest edge will suffer damage. Such quantifying of risk on a fine scale in this way can be useful for wildlife managers seeking to persuade local farmers to change crops. Knowledge sharing with local communities is critically important [52], particularly in the case of large carnivore conservation where excessive fear exists, and our visualized maps are likely to be helpful in attempts to change local attitudes.

Our findings indicate that red pine forest is the main reason for bear occurrence in peripheral human lands in our region. It is, however, not practical and against conservation practices to eradicate all red pine forests in order to drive bears from the foothills. Traditional coppice forest (called *Satoyama* in Japanese) management has been shown to be beneficial to species diversity [9], insofar as it entails only intermediate levels of human disturbance. For this reason, restoration of *Satoyama* management of red pine forest is probably the most suitable way of creating buffer structures without the removal of the critical summer habitat of Asiatic black bears. At a broader scale, large areas of plantation are left unmanaged, resulting in increased canopy closure and a dramatic reduction of open regenerating lands and edge habitat in mountain areas. We expect that the creation of open lands in unmanaged plantation, by providing a suitable summer habitat for bears in areas isolated from human landscape, would provide a new opportunity to reduce human-bear interaction.

The rapid increase in human-wildlife interaction in Japan against a background of an aged and de-populated rural society, and the abandonment of forestry and agricultural practices, may be an exceptional case in global terms. Although the demographic process is the opposite of that found in nations of rapid human population growth, the problems caused by the sharpening of the human-wildlife boundaries are similar. As many researchers have recognized, fencing is not a panacea [53], and the creation of distance between large carnivores and humans would be a more sophisticated approach to long-term mitigation. One example of this in our case would be the restoration of season-specific habitats in remote areas. We conclude that a habitat-based approach has enormous potential as a means of creating adequate separation between humans and wildlife. The results of our study imply that human land use has an indirect but fundamental influence on the frequency of human-wildlife interaction. However, this linkage has rarely been investigated. Thus, we recommend that further research be undertaken to understand the mechanisms of increased human-wildlife interaction in relation to the influence of anthropogenic land modification and management processes. We believe this would provide a new direction in the search for ways to achieve co-existence with large carnivores by resolving the complex issue of conflict.

## Supporting Information

Table S1 **Logistic regression coefficients, odds ratio and 95% Confidence Interval for the odds ratio estimated by the final habitat selection model on the basis of mixed effect logistic regression for each season-sex group of Asiatic black bears.** *ß* was a coefficient and LRT indicated Log-likelihood Ratio Test in comparison with the constant only model. n denoted were the number of observations. The odds ratios and their 95% CI were given as 10^5^ (denoted ^a^) and 10^2^ (denoted ^b^) times the original value of their coefficients.
(XLS)

Table S2 **Estimated coefficients for RSF models of habitat selection by Asiatic black bears; averaged coefficients (mean** ***ß*** **) estimated by individual level, and coefficients (** ***ß*** ), standard errors (S.E.), and significance (* = p<0.01) estimated by population level (each sex-season group) in the mixed effect logistic regressions.
(XLS)
